# Association of cardiometabolic multimorbidity with postoperative delirium and three-year mortality in patients undergoing knee/hip arthroplasty: a prospective cohort study

**DOI:** 10.1097/JS9.0000000000002379

**Published:** 2025-05-28

**Authors:** Kun Wang, Aihua Zhang, Wenjie Kong, Yuanlong Wang, Yizhi Liang, Yanan Lin, Chuan Li, Jiahan Wang, Hongyan Gong, Yanlin Bi, Bin Wang, Xu Lin

**Affiliations:** a Department of Anesthesiology, Qingdao Municipal Hospital, Qingdao, Shandong, China; b School of Anesthesiology, Shandong Second Medical University, Weifang, China; c The Second School of Clinical Medicine of Binzhou Medical University, Yantai, China

**Keywords:** anesthesiology, cardiometabolic multimorbidity, cerebrospinal fluid, cohort study, delirium, neuropsychological tests, postoperative delirium

## Abstract

**Introduction:**

Postoperative delirium (POD) is a severe and common complication. This study aimed to investigate the association of cardiometabolic multimorbidity (CMM) and their different subgroups with POD.

**Methods:**

This prospective cohort study ultimately included 875 patient samples from the Perioperative Neurocognitive Disorder and Lifestyle Biomarkers (PNDABLE) database, collected between July 2020 and September 2021. In this study, patients were first categorized into a POD group and a non-POD group, and the demographic characteristics of the two groups were compared. Next, logistic regression models were used to analyze the association between CMM and POD, as well as between cerebrospinal fluid (CSF) biomarkers and POD. Additionally, the models examined the relationship between different CMM subtypes and the incidence of POD. Subsequently, the robustness of the results was verified by sensitivity analysis and post hoc analysis. Further, the role of CSF biomarkers in the relationship between CMM and POD was assessed using mediation analysis. Finally, CMM patients with POD were followed up for three years, and Kaplan–Meier (K-M) survival analysis was used to compare the mortality rates of different CMM subgroups in patients with POD.

**Results:**

Logistic regression analysis showed that CMM [odds ratio: 5.062; 95% CI: 3.279–7.661; *P* < 0.001], T-tau, and P-tau were risk factors for POD, while Aβ42 was a protective factor. Associations between different CMM subgroups and POD varied. Sensitivity and post hoc analyses supported these findings. Mediation analysis indicated that CMM could increase the incidence of POD through the CSF T-tau (proportion: 11%, *P* < 0.050). A follow-up of 50 patients showed that K-M survival analysis revealed that the POD patients in the diabetes combined with coronary heart disease group had a significantly higher three-year mortality compared to other CMM subgroups (*P* = 0.004).

**Conclusions:**

CMM may be a risk factor for POD, with CSF T-tau potentially playing a mediating role. These findings underscore the importance of preoperative cognitive assessment for risk stratification and suggest CSF T-tau as a potential intervention target. Future studies may further explore intervention strategies targeting CMM and CSF T-tau.

**HIGHLIGHTS**

- Cardiometabolic multimorbidity (CMM) is independently associated with an increased risk of postoperative delirium (POD).
- Cerebrospinal fluid T-tau mediated the association between CMM and POD.
- The subgroup of POD patients with diabetes combined with coronary heart disease had the highest three-year mortality.
- Targeted interventions may reduce the incidence of POD and associated mortality rates.

## Introduction

Postoperative delirium (POD) is an acute brain dysfunction that affects approximately 10-50% of surgical patients, characterized by fluctuating attention, cognition, and consciousness^[^1,2^]^. POD often occurs within 1–3 days after surgery, and its main manifestations include cognitive dysfunction, decreased level of consciousness, inability to concentrate, decreased mental activity, and disruption of the sleep-wake cycle^[^2,3^]^. It is associated with poorer functional recovery, longer intensive care stays, and increased mortality^[^4^]^. Current research hypotheses for POD include the blood-brain barrier injury theory, the cholinergic neural theory, and the Aβ protein deposition and tau protein phosphorylation theories; however, the exact pathogenesis of POD remains unclear, and key questions that need to be addressed urgently include the interrelationships between these theories and how they contribute to the development of postoperative cognitive dysfunction^[^5^]^. Among them, the Aβ protein deposition and tau protein phosphorylation theory has received the widest support^[^6^]^. Studies have shown that neuronal damage plays a vital role in the pathogenesis of POD, and cerebrospinal fluid biomarkers can reflect this damage^[^7^]^. In our previous research and other relevant literature, a close association has been established between Aβ42, total tau protein (T-tau), phosphorylated tau protein (P-tau), and the incidence of POD^[^8,9^]^. For example, one study reported that lower levels of Aβ42 in cerebrospinal fluid (CSF), which may reflect increased amyloid plaques in the brain, are associated with an elevated risk of POD^[^10^]^. Similarly, elevated levels of T-tau, a marker of axonal injury and neuronal death, have been observed in patients who develop POD, suggesting the presence of acute neuronal injury in the perioperative period^[^11^]^.

Cardiometabolic multimorbidity (CMM) refers to the coexistence of two or more cardiometabolic diseases (CMD), including hypertension, diabetes, coronary heart disease (CHD), and stroke^[^12^]^. With an increasingly aging population, 4.7% of middle-aged and elderly patients now have at least two cardiometabolic diseases^[^13^]^. CMM is strongly associated with decreased function, increased healthcare costs, and higher mortality^[^14^]^. In addition, there is growing evidence of an association between CMM and cognitive dysfunction^[^14-16^]^.

Although the association between CMM and POD has garnered considerable attention, the specific relationship and underlying mechanisms remain unclear. In particular, hypertension may lead to cerebrovascular damage, diabetes may cause neuronal damage and inflammatory responses, CHD may lead to cerebral microangiopathy, and stroke may lead directly to brain damage. Common to these diseases are chronic inflammation, endothelial dysfunction, and metabolic disorders, which may act synergistically to accelerate neurodegenerative processes^[^10,17,18^]^. And it has been suggested in the literature that the confluence of chronic inflammation, endothelial dysfunction, and metabolic disorders in CMM may synergistically accelerate neurodegenerative processes through mechanisms involving increased Aβ production or decreased clearance and enhanced tau protein phosphorylation^[^19^]^.

A study by Glumac demonstrated that POD is a strong predictor of postoperative cognitive decline (POCD), and that there are significant differences between POCD and POD in terms of timing and impact. In addition, there is a growing body of evidence supporting the association between CMM and cognitive dysfunction^[^20^]^. This study, based on population samples, aims to achieve the following objectives: (1) to explore the relationships among CMM, POD, and CSF biomarkers in the Han Chinese population; (2) to investigate the potential mediating effects of CSF biomarkers in the relationship between CMM and POD; (3) to analyze the associations between different subgroups of CMM and POD; (4) to examine the impact of different CMM subgroups on the three-year mortality of POD patients.

We hypothesized that there is a significant association between CMM and POD and that CSF biomarkers mediate this relationship. In addition, we hypothesized that different CMM subgroups would have different effects on three-year mortality in patients with POD.

This cohort/cross-sectional/case-control study has been reported in line with the STROCSS guidelines.

## Methods

### PNDABLE database

The “Perioperative Neurocognitive Disorder and Biomarkers of Lifestyle (PNDABLE)” database, a long-term independent cohort study, provided the data for this investigation into risk factors and biomarkers associated with perioperative neurocognitive disorders in the Han Chinese population.

Participants, aged 40–90 years, were recruited from the large tertiary hospital. All participants provided voluntary informed consent and retained the right to withdraw from the study at any time. The PNDABLE study, adhering to the ethical principles outlined in the Declaration of Helsinki, has received ethical approval from the Ethics Committee of Hospital and is registered with the China Clinical Trial Registry.

### Eligibility criteria

Data for this prospective cohort study were obtained from the PNDABLE database, covering the period between July 2020 and September 2021. Participants were selected based on the following criteria: (1) aged 40–90 years, (2) scheduled for knee or hip replacement surgery, (3) able to complete neuropsychiatric evaluations independently, (4) classified as grade I-III by the American Society of Anesthesiologists (ASA), and (5) receiving combined spinal-epidural anesthesia.

Exclusion criteria included: (1) Patients scoring below 24 on the pre-surgical Mini-Mental State Examination (MMSE); (2) a history of substance or psychotropic drug abuse or prolonged use of steroids or hormonal treatments; (3) significant visual or hearing impairments; (4) severe systemic illnesses (e.g., cancer) that could impact CSF biomarker levels; (5) serious psychiatric conditions; or (6) disorders affecting speech or communication.

### Clinical evaluation and covariates

CMD, which included hypertension, diabetes, CHD, and stroke, was diagnosed using a standardized approach that combined patient self-reports, medication records, and electronic medical record (EMR) diagnoses, ensuring the accuracy and rigor of the diagnosis through a multi-source verification process. Patients with two or more concurrent CMDs are defined as having CMM. Based on CMD status, CMM patients are further categorized into seven groups: hypertension combined with diabetes, hypertension combined with CHD, hypertension combined with stroke, diabetes combined with CHD, CHD combined with stroke, patients with three CMDs, and patients with four CMDs, as part of the supplementary study.

Basic information included age (continuity), sex (female vs. male), years of education (continuity), MMSE score (continuity), height (continuity), weight (continuity), infusion volume (continuity), duration of surgery (continuity), duration of anesthesia (continuity), ASA classification (class I, II vs. III), body mass index (BMI: continuity), smoking status (nonsmoker vs. current/former smoker), alcohol consumption [non-drinker/occasional drinker (at least once a month but less than once a week) vs. regular drinker], and physical activity [active (at least 150 minutes of moderate-intensity or 75 minutes of vigorous-intensity physical activity per week) vs. inactive (did not meet the above criteria)].

### Anesthesia and surgery

Patients undergoing hip or knee replacement surgery received spinal and epidural anesthesia. A 6-8 hour fast and fluid restriction preceded the procedure. Upon arrival in the operating room, standard monitoring (electrocardiography, oxygen saturation, arterial blood pressure, and venous access) was initiated.

A lumbar puncture at the L3–L4 intervertebral space yielded 2 ml of CSF for biomarker analysis. This was followed by the injection of 2–2.5 ml of 0.66% ropivacaine over 30 seconds. Anesthesia was maintained below the T8 level throughout surgery, with vital signs (blood pressure, heart rate, and oxygen saturation) monitored every 3 minutes. Oxygen was delivered at 5 liters per minute via mask. Post-operatively, patients were observed in the PACU for 30 minutes. Upon stabilization, they were transferred back to the ward.

### Collection and measurement of CSF biomarkers

Two milliliters of CSF were collected in a polypropylene centrifuge tube. The sample was then centrifuged at 2000 × g for 10 minutes at room temperature, and the supernatant was transferred to an enzyme-free Eppendorf tube (PCR02-C oxygen bottle) before being stored at −80°C for subsequent analyses.

Enzyme-linked immunosorbent assay (ELISA) was employed to quantitatively determine the concentrations of Aβ42, T-tau, and P-tau in 2 ml of CSF, using commercially available assay kits (Thermo Scientific, Multiskan MK3) according to the manufacturer’s protocols. The ratios of Aβ42/T-tau and Aβ42/P-tau were subsequently calculated. All measurements were conducted by the same laboratory personnel with the group assignments blinded to ensure objectivity.

### Neuropsychological testing

Preoperatively, the neurologist assessed the patient’s cognitive function using the MMSE and excluded patients with MMSE scores below 24. The delirium assessment was performed two times a day at 9:00–10:00 am and 2:00–3:00 pm on 1–7 days (or before discharge) by an anesthesiologist postoperatively, and the diagnosis of POD was made using the Confusion Assessment Method (CAM), which was assessed daily on postoperative days 1 through 7 or before patient discharge. Patients with positive test results were categorized into the POD group, and those with negative results were categorized into the non-POD (NPOD) group. Both the MMSE and the CAM are commonly used and reliable tools for assessing the cognitive status of patients^[^21,22^]^.

### Follow-up

Survival time was measured from the date of surgery to either the occurrence of death or the most recent follow-up appointment. Investigators followed patients with CMM coexisting with POD for three years postoperatively.

### Statistical analysis

Data analysis began with the Kolmogorov-Smirnov test to assess the normality of continuous variables. Normally distributed variables were expressed as mean ± standard deviation (SD), while non-normally distributed variables were summarized as median and interquartile range (M, Q25, Q75). Group comparisons for continuous variables utilized either the independent samples t-test or the Mann–Whitney U test, based on data distribution. Categorical variable differences between groups were determined using the χ^2^ test or Fisher’s exact test.

Logistic regression analysis was used to investigate whether CMM and CSF biomarkers independently affect POD and whether they are risk factors or protective factors for POD. To enhance the transparency and interpretability of variable selection, we constructed a directed acyclic graph (DAG). All candidate variables for the multivariate model were entered simultaneously using the Enter method to control for potential covariances between variables. To reduce the risk of potential multicollinearity, we performed a variance inflation factor (VIF) analysis to examine the potential shared variance among the variables. The robustness of the results was subsequently verified by a two-step sensitivity analysis (1) adjusting for the underlying confounders, including sex, years of education, BMI, and MMSE, and (2) further extending the set of adjusted variables to incorporate smoking history, alcohol history, duration of anesthesia, and physical activity in order to assess the interference of potential confounders. In addition, the effects of different CMM subgroups on POD were further analyzed. One-way logistic regression was first performed, and subgroups with significance in the one-way regression were continued to subsequent multifactorial logistic regression and sensitivity analyses. Given that multiple comparisons may increase the risk of type I error, the Bonferroni method was used to correct the *P* values with a correction threshold of α/n (n is the number of independent tests).

Additionally, the mediating role of CSF biomarkers in the association between CMM and POD was examined using the Bootstrap method. To reduce the risk of potential multicollinearity, CSF biomarkers [Aβ42, T-tau, P-tau, Aβ42/T-tau, Ab42/P-tau] were centered before analysis. Five independent mediation models were constructed with CMM as the independent variable, POD as the dependent variable, and each CSF biomarker after centering as the mediator variable. The mediating effects were assessed based on the following four criteria: (1) the association between CMM and POD was attenuated after CSF biomarkers were included in the model; (2) CSF biomarkers were significantly associated with POD; (3) CMM was significantly associated with POD; and (4) CMM was significantly associated with CSF biomarkers.

Post hoc analysis was performed, excluding patients with MMSE <28, smoking, and BMI ≥30, to explore whether the study results are applicable to individuals with better cognitive function, non-smokers, and non-obese individuals.

Finally, Kaplan–Meier (K-M) survival analysis was performed to assess the effect of different CMM subgroups on the three-year mortality in POD patients, with the Log-rank test used to evaluate survival risk differences between groups.

Statistical analyses were conducted using a combination of statistical software packages, including SPSS, R, and Stata. Statistical significance was set at *P* ≤ 0.050.

### Sample size estimation

Eight covariates were assessed in preliminary analyses to inform the development of the logistic regression model. Previous studies suggest a POD prevalence of approximately 10%, and a 20% loss to follow-up was anticipated. Based on logistic regression event per variable (EPV) sample size calculations, with an EPV of 10 and a desired power of 80%, a sample size of 1000 cases was determined (1000 = 8 × 10 ÷ 0.10 ÷ 0.8)^[^23^]^.

### Statement

The work has been reported in line with the STROCSS criteria^[^24^]^.

## Results

### Patient screening and grouping

**Figure 1.**
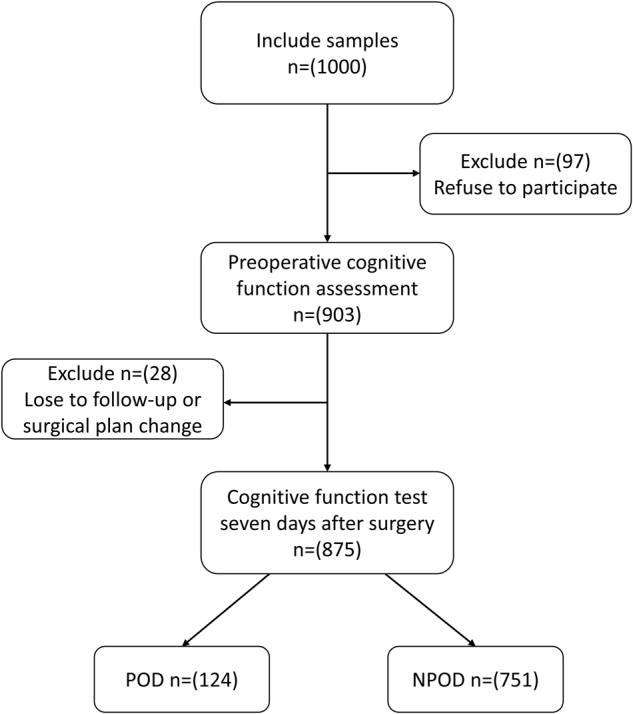
The flow diagram showed the selection of eligible patients and the enrollment process. POD: postoperative delirium; NPOD: no postoperative delirium.

A total of 1000 patients were initially screened for the study. After excluding 125 patients who did not meet the inclusion criteria, 875 patients with complete clinical data were included in this retrospective study. These patients were then categorized into the POD (124 patients) and NPOD (751 patients) groups. Figure 1 outlines how patients were screened, with 125 excluded based on predefined criteria.

### Clinical characteristics of the patients

Of the 875 patients ultimately included, 124 patients developed POD, resulting in a POD prevalence of 14.2 %.

In the POD group, patients were older (*P* < 0.001), had a higher proportion of physical activity (*P* = 0.041), and a greater prevalence of hypertension, diabetes, CHD, stroke, and CMM compared to those in the NPOD group (*P* < 0.001). CSF biomarkers, including Ab42, T-tau, P-tau, Ab42/T-tau, and Ab42/P-tau, showed significant differences between the groups (*P* < 0.001), with elevated Ab42 and decreased T-tau and P-tau levels in the NPOD group.

No notable differences were observed between the two groups in terms of gender, height, weight, BMI, years of education, duration of surgery, amount of intraoperative fluids, duration of anesthesia, history of smoking, and history of alcohol consumption. For detailed information, see Table 1.

**Table 1:** Demographic and clinical characteristics of participants

	NPOD	POD	*P*
Number	751	124	
Age, M (IQR), years	61(54–67)	74(71–79)	**<0.001**
Height, M (IQR), cm	168(160–173)	166(161–172)	0.329
Weight, M (IQR), kg	70(63–79)	70(61–78)	0.603
BMI, M (IQR)	25.32(23.03–27.41)	25.02(22.78–28.07)	0.970
Education, M (IQR), year	9(8–12)	9(6–12)	0.703
MMES, score, M (IQR)	28(27–29)	28(27–29)	0.115
Volume of fluid, M (IQR), ml	1100(600–1100)	1000(600–1100)	0.137
Duration of surgical, M (IQR), h	1.56(0.92–2.00)	1.56(1.02–2.08)	0.727
ASA classification (%)			**<0.001**
I	87(11.6)	1(0.8)	
II	634(84.4)	98(79.0)	
III	30(4.0)	25(20.2)	
Sex (female/male)	316/435	49/75	0.592
Duration of anesthesia, M(IQR), h	2.40(1.67–3.00)	2.33(1.83–2.93)	0.875
Hypertension, yes (%)	250(33.3)	73(58.4)	**<0.001**
Diabetes, yes (%)	96(12.8)	45(36.0)	**<0.001**
CHD, yes (%)	62(8.3)	36(28.8)	**<0.001**
Stroke, yes (%)	27(3.6)	14(11.2)	**<0.001**
Cigarette smoking, yes (%)	216(28.8)	30(24.0)	0.295
Drinking, yes (%)	255(34.0)	35(28.0)	0.209
Physical activity, yes (%)	231(30.8)	53(42.4)	**0.041**
CMM, yes (%)	89(11.9)	50(40.0)	**<0.001**
CSF biomarkers, M (IQR), pg/mL			
Aβ42	412.74(284.01–573.66)	244.99(144.65–405.90)	**<0.001**
T-tau	186.60(143.84–255.20)	276.74(205.40–589.56)	**<0.001**
P-tau	39.12(31.62–50.47)	61.27(41.04–82.47)	**<0.001**
Ratios of biomarkers, M (IQR)			
Aβ42/T-tau	2.19(1.42–3.25)	0.96(0.35–1.70)	**<0.001**
Aβ42/P-tau	10.37(6.89–15.21)	4.66(2.09–8.30)	**<0.001**

Categorical variables are reported as numbers and percentages; continuous variables are reported as means ± SD, whereas non-normal data are expressed as the M (Q25, Q75).

Abbreviations: POD: postoperative delirium; NPOD: no postoperative delirium; M: median; IQR: interquartile range; BMI: body mass index; MMSE: minimum mental state examination; ASA: American Society of Anesthesiologists; CHD: coronary heart disease; CMD: cardiometabolic diseases.

The bold values are all *P* ≤ 0.050, statistically significant parameters.

### Logistic regression and sensitivity analysis of risk and protective factors for POD

To investigate the risk and protective factors for POD, we first constructed a directed acyclic graph (DAG) to enhance the transparency of our variable selection; the results of the DAG are presented in Supplementary Figure 1 (available at: http://links.lww.com/JS9/E54). We then conducted univariate logistic regression analyses by including CMM, Aβ42, T-tau, P-tau, Aβ42/P-tau, and Aβ42/T-tau. The results indicated that CMM (OR, 5.026; 95% CI: 3.297–7.661; *P* < 0.001), T-tau (OR, 1.007; 95% CI: 1.005–1.008; *P* < 0.001), and P-tau (OR, 1.058; 95% CI: 1.047–1.070; *P* < 0.001) were significant risk factors for POD, while Aβ42 (OR, 0.995; 95% CI: 0.994–0.997; *P* < 0.001), Aβ42/T-tau (OR, 0.292; 95% CI: 0.223–0.382; *P* < 0.001), and Aβ42/P-tau (OR, 0.775; 95% CI: 0.732–0.821; *P* < 0.001) were protective factors.

To further control for potential confounding factors, we incorporated the CSF biomarkers (Aβ42, T-tau, P-tau, Aβ42/P-tau, and Aβ42/T-tau) along with confounding variables (ASA and stroke) into a multivariate logistic regression model. In the collinearity analysis, the VIF values of the confounding factors were all less than 10, indicating that there is no collinearity among these confounding factors, and the specific results are shown in Supplementary Table I (available at: http://links.lww.com/JS9/E55)^[^25^]^. The outcomes suggested that CMM (OR, 3.859; 95% CI: 2.188–6.805; *P* < 0.001), T-tau (OR, 1.003; 95% CI: 1.001–1.005; *P* = 0.006), and P-tau (OR, 1.049; 95% CI: 1.028–1.070; *P* < 0.001) remained significant risk factors for POD, while Aβ42 (OR, 0.997; 95% CI: 0.993–1.000; *P* = 0.027) was a protective factor. However, Aβ42/T-tau (OR, 0.785; 95% CI: 0.459–1.343; *P* = 0.377) and Aβ42/P-tau (OR, 1.041; 95% CI: 0.942–1.149; *P* = 0.431) were not statistically significant.

Following two rounds of sensitivity analysis, our results remained robust, with T-tau, and P-tau continuing to show statistical significance. Moreover, as demonstrated, CMM remain a significant risk factor for POD (OR, 2.984; 95% CI: 1.478–6.022; *P* = 0.002) (Table 2).

**Table 2:** Logistic regression and sensitivity analysis in PNDABLE

	Model 1		Model 2		Model 3		Model 4	
	OR	*P*	OR	*P*	OR	*P*	OR	*P*
	(95%CI)						(95%CI)	
			(95%CI)		(95%CI)			
CMM	5.026	**<0.001**	3.859	**<0.001**	3.840	**<0.001**	2.984	**0.002**
	(3.297–7.661)		(2.188–6.805)		(2.154–6.846)		(1.478–6.022)	
Aβ42	0.995	**<0.001**	0.997	**0.027**	0.997	**0.036**	0.997	0.151
	(0.994–0.997)		(0.993–1.000)		(0.994–1.000)		(0.994–1.001)	
T-tau	1.007	**<0.001**	1.003	**0.006**	1.003	**0.005**	1.003	**0.007**
	(1.005–1.008)		(1.001–1.005)		(1.001–1.006)		(1.001–1.006)	
P-tau	1.058	**<0.001**	1.049	**<0.001**	1.049	**<0.001**	1.036	**0.004**
	(1.047–1.070)		(1.028–1.070)		(1.028–1.070)		(1.012–1.061)	
Aβ42/T-tau	0.292	**<0.001**	0.785	0.377	0.780	0.371	0.826	0.538
	(0.223–0.382)		(0.459–1.343)		(0.452–1.345)		(0.450–1.518)	
Aβ42/P-tau	0.775	**<0.001**	1.041	0.431	1.036	0.490	0.999	0.990
	(0.732–0.821)		(0.942–1.149)		(0.937–1.146)		(0.900–1.109)	

OR, odds ratio; 95% CI, 95% confidence interval.

Model 1: the unadjusted logistic regression.

Model 2: adjusted logistic regression.

Model 3: first sensitivity analysis was based on more covariates including years of education, BMI, and MMSE.

Model 4: second sensitivity analysis was based on more covariates including years of education, BMI, MMSE, sex, smoking history, alcohol history, and duration of anesthesia.

The bold values are all *P* ≤ 0.050, statistically significant parameters.

CMM: cardiometabolic multimorbidity

### Logistic regression and sensitivity analysis for different subgroups of CMM

To explore the relationship between different subgroups of CMM and POD, we divided the patients into seven subgroups: hypertension combined with diabetes, hypertension combined with CHD, hypertension combined with stroke, diabetes combined with CHD, CHD combined with stroke, simultaneous combination of three CMDs, and simultaneous combination of four CMDs. The results of univariate logistic regression analysis showed that hypertension combined with diabetes (OR, 3.581; 95% CI: 1.952–6.568; *P* < 0.001; *P-adj* < 0.007), diabetes combined with CHD (OR, 6.269; 95% CI: 1.788–21.981; *P* = 0.004; *P*-adj = 0.028) and the simultaneous combination of three CMDs (OR, 37.213; 95% CI: 2.995–17.372; *P* < 0.001; *P-adj* < 0.007) were all significant risk factors for POD.

Subsequently, we included these subgroups in a multivariate logistic regression model for further analysis. The analysis revealed that hypertension combined with diabetes (OR, 3.718; 95% CI: 1.767–7.827; *P* < 0.001; *P-adj* < 0.003), diabetes combined with CHD (OR, 6.385; 95% CI: 1.041–39.174; *P* = 0.013; *P-adj* = 0.039) and the three comorbidities group (OR, 3.562; 95% CI: 1.189–10.670; *P* = 0.010; *P-adj* = 0.030) remained significant independent risk factors for POD.

After two rounds of sensitivity analysis, the results further confirmed that hypertension combined with diabetes and the simultaneous combination of three CMDs remained significant risk factors for POD (Table 3).

**Table 3:** Logistic regression analysis and sensitivity analysis for different subgroups of CMM

	MODEL1			MODEL2			MODEL3			MODEL4		
	OR(95%CI)	*P*	*P-adj*	OR(95%CI)	*P*	*P-adj*	OR(95%CI)	*P*	*P-adj*	OR(95%CI)	*P*	*P-adj*
A	3.581	**<0.001**	**<0.007**	3.673	**<0.001**	**<0.003**	3.800	**<0.001**	**<0.003**	2.747	**0.009**	**0.027**
	(1.952–6.568)			(1.968–6.858)			(2.019–7.151)			(1.286–5.868)		
B	2.182	0.051	-	-	-	-	-	-	-	-	-	-
	(0.997–4.775)			-			-			-		
C	1.352	0.702	-	-	-	-	-	-	-	-	-	-
	(0.289–6.330)			-			-			-		
D	6.269	**0.004**	**0.028**	5.237	**0.013**	**0.039**	5.362	**0.012**	**0.036**	3.081	0.157	-
	(1.788–21.981)			(1.418–19.343)			(1.441–19.946)			(0.648–14.651)		
E	12.295	**0.041**	0.287	-	-	-	-	-	-	-	-	-
	(1.106–136.629)			-			-			-		
F	7.213	**<0.001**	**<0.007**	3.717	**0.010**	**0.030**	3.586	**0.013**	**0.039**	6.015	**0.008**	**0.024**
	(2.995–17.372)			(1.370–10.080)			(1.304–9.859)			(1.584–22.840)		
G	3.061	0.199	-	-	-	-	-	-	-	-	-	-
	(0.555–16.895)			-			-			-		

A: hypertension combined with diabetes.

B: hypertension combined with CHD.

C: hypertension combined with stroke.

D: diabetes combined with CHD.

E: CHD combined with stroke.

F: simultaneous combination of three CMDs.

G: simultaneous combination of four CMDs.

Model 1: the unadjusted logistic regression.

Model 2: adjusted more covariates logistic regression including Ab42, T-tau, P-tau, Ab42/T-tau, Ab 42/P-tau, hypertension and stroke.

Model 3: first sensitivity analysis was based on more covariates including years of education, BMI, and MMSE.

Model 4: second sensitivity analysis was based on more covariates including years of education, BMI, MMSE, sex, smoking history, alcohol history, and duration of anesthesia.

The bold values are all *P* ≤ 0.050, statistically significant parameter.

### Mediation analysis

**Figure 2.**
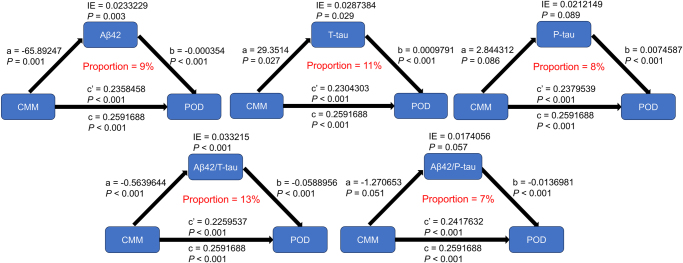
Mediation analyses with 10 000 bootstrapped iterations were used to examine the mediation effects of Ab42, T-tau, P-tau, Ab42/T-tau, and Ab42/P-tau on POD. POD: postoperative delirium; CMM: cardiometabolic multimorbidity.

Mediation analysis showed that CMM could increase the prevalence of POD via CSF biomarkers, including T-tau (proportion: 11%, *P* < 0.050) and Ab42/T-tau (proportion: 13%, *P* < 0.050). See Fig. 2 for details.

### Post hoc analyses

The PNDABLE cohort was studied in after excluding a portion of the MMSE<28, the smoking population, and the obese population. The findings were as follows: model 1 [CMM (OR,4.224; 95% CI: 1.994–8.947; *P* < 0.001)]; model 2 [CMM (OR,3.243; 95% CI: 1.606–6.549; *P* = 0.001)]; model 3 [CMM (OR,3.668; 95% CI: 1.966–6.843; *P* < 0.001)], indicating that our results remained stable, suggesting that CMM as a risk factor for POD (Table 4).

**Table 4:** Post hoc analyses in the PNDABLE study

	Model 1		Model 2		Model 3	
	OR	*P*	OR	*P*	OR	*P*
	(95%CI)		(95%CI)		(95%CI)	
CMM	4.224	**<0.001**	3.243	**0.001**	3.668	**<0.001**
	(1.994–8.947)		(1.606–6.549)		(1.966–6.843)	
Aβ42	0.994	**0.004**	0.996	**0.018**	0.996	**0.038**
	(0.990–0.998)		(0.993–0.999)		(0.993–1.000)	
T-tau	1.005	**0.004**	1.003	**0.016**	1.003	**0.010**
	(1.002–1.008)		(1.001–1.005)		(1.001–1.006)	
P-tau	1.060	**<0.001**	1.053	**<0.001**	1.048	**<0.001**
	(1.035–1.087)		(1.029–1.077)		(1.026–1.070)	
Aβ42/T-tau	0.973	0.934	0.705	0.271	0.697	0.247
	(0.511–1.855)		(0.379–1.313)		(0.379–1.283)	
Aβ42/P-tau	1.097	0.084	1.088	0.088	1.050	0.358
	(0.988–1.218)		(0.988–1.199)		(0.947–1.164)	

Model 1: exclude MMSE < 28.

Model 2: exclude cigarette smoking.

Model 3: exclude BMI ≥ 30.

The bold values are all *P* ≤ 0.050, statistically significant parameters. Model 1–3 refers to the model of post hoc analyses.

CMM: cardiometabolic multimorbidity

### Survival curve construction based on CMM subgroups for postoperative follow-up of POD patients

This study followed 50 CMM patients who developed POD for three years, with 7 patients deceased and no loss to follow-up, resulting in an overall survival rate of 86%.

To assess the differences in three-year mortality between CMM subgroups significantly and non-significantly associated with POD, we classified the CMM subgroups that were non-significantly associated with POD in the subgroup analysis as the “POD non-significantly associated subgroup,” which included hypertension combined with CHD, hypertension combined with stroke, CHD combined with stroke, and the four diseases of CMDs. This subgroup was then compared with hypertension combined with diabetes group, diabetes combined with CHD group, and three diseases of CMDs using K-M survival analysis.

The three-year mortality for each subgroup was as follows: POD non-significantly associated subgroup 6.25% (1/16), hypertension combined with diabetes group 11.1% (2/18), diabetes combined with CHD group 60% (3/5), and three diseases of CMDs 9.1% (1/11). The three-year mortality risk was significantly elevated (*P* = 0.004) in the cohort presenting with comorbid diabetes and CHD compared to all other groups.

**Figure 3.**
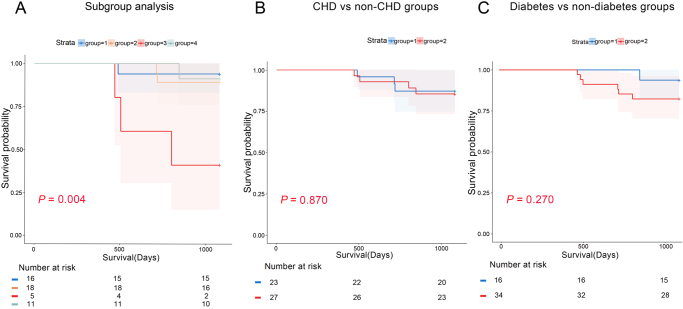
Survival analysis of POD patients in different subgroups. (A) Group 1: POD non-significantly associated subgroup; Group 2: hypertension combined with diabetes; Group 3: diabetes combined with CHD; Group 4: three diseases of CMDs. (B) Group 1: diabetes-free; Group 2: diabetes. (C) Group 1: CHD-free; Group 2: CHD POD: postoperative delirium; CHD: coronary heart disease; CMD: cardiometabolic diseases.

To further explore the underlying mechanisms of this result, we stratified patients based on the presence or absence of diabetes and CHD. The results showed no significant differences in three-year survival rates between the diabetes and non-diabetes groups or between the CHD and non-CHD groups (*P* values of 0.270 and 0.870, respectively) (Fig. 3).

## Discussion

This prospective cohort study is the first to identify CMM as a risk factor for POD, with T-tau playing a mediating role in this relationship. Additionally, among POD patients with CMM, those with comorbid diabetes and CHD exhibited the highest three-year mortality rate.

For the first time, the study has identified CMM as an independent risk factor for POD, with this relationship partially mediated by biomarkers in CSF, particularly the elevated levels of T-tau. The research also demonstrated that elevated Aβ42 is a protective factor against POD. In contrast, elevated P-tau and T-tau proteins are risk factors for POD, which is consistent with our previous research findings^[^26-28^]^. Tau protein is a microtubule-associated protein within neurons, and during neuronal damage, tau is released into the CSF from damaged neurons^[^29,30^]^. The mediation of POD through elevated T-tau levels suggests that CMM-induced cerebrovascular and neuronal damage may be a core mechanism. This finding is similar to the results obtained by Dr. Maria Vassilaki, who found a cross-sectional association between cardiometabolic disturbances, multimorbidity, and reduced cortical thickness in specific regions but without amyloid accumulation in the brain^[^31^]^. Dr. Xin You Tai also reported that cardiometabolic multimorbidity is associated with hippocampal and gray matter atrophy^[^32^]^. These studies support the hypothesis that cerebrovascular and neuronal damage may play a key role in linking CMM with POD.

The subgroup analysis revealed that different types of CMM have varying effects on the risk of POD, with a significantly elevated POD risk observed in patients with hypertension combined with diabetes, diabetes combined with CHD, and those with three CMDs. The underlying mechanism may be related to the more severe cerebral blood flow impairment and inflammatory responses caused by these comorbidities^[^13,33^]^. For instance, the combined effect of diabetes and CHD exacerbates macrovascular and microvascular damage, which may accelerate neurodegenerative processes^[^34-36^]^. The coexistence of three CMDs likely amplifies the risk of POD through synergistic mechanisms, including cumulative pro-inflammatory cytokine release and extensive vascular injury^[^37,38^]^. These findings suggest that personalized intervention strategies tailored to the specific pathological characteristics of each subgroup are necessary to mitigate the risk of POD effectively.

Through follow-up analysis, we found for the first time that POD patients in the diabetes combined with CHD group had the highest three-year mortality. Studies have shown that the comorbidity of diabetes and CHD not only leads to more extensive vascular lesions but may also exacerbate brain injury by affecting cerebral blood flow^[^34^]^. Additionally, these patients often have metabolic abnormalities and dysregulated inflammatory responses, making them more susceptible to postoperative complications such as malignant arrhythmias and acute heart failure, thereby increasing the risk of death^[^39,40^]^. Therefore, the observed result may be attributed to the simultaneous presence of these two conditions, which is associated with a higher incidence of cardiovascular events and more severe vascular damage. Special attention should be given to cardiovascular protection and nutritional and metabolic support in the postoperative care of this high-risk group.

Previous studies have indicated that either diabetes or CHD alone can increase mortality; however, there has been little exploration of the combined impact of these conditions on the mortality rate of POD patients^[^41,42^]^. The results of this study showed that diabetes or CHD alone did not significantly increase mortality in patients with POD in the presence of CMM. This suggests that although diabetes and CHD independently have significant adverse effects on the cardiovascular system, their independent effects may not be sufficient to significantly alter survival outcomes in POD patients when multiple comorbidities are present^[^43,44^]^. This finding also suggests that the coexistence of diabetes and CHD may impact prognosis through more complex mechanisms within specific pathological contexts. In other words, the combined effects of diabetes and CHD may lead to a more intricate physiological impact by contributing to widespread vascular damage, metabolic disturbances, and systemic inflammatory responses, especially during the perioperative period in POD patients^[^45^]^.

This study had certain limitations. First, despite the relatively large overall sample size, some of the subgroups had small sample sizes, a limitation that may have diminished our ability to detect statistically significant associations between these subgroups and POD. Also, our study sample included only one patient with diabetes combined with stroke, and this underrepresentation limited our in-depth analysis of the association between this subgroup and POD, which in turn affected the comprehensiveness of the subgroup analysis. In addition, when performing the K-M survival analysis, only patients with CMM combined with POD were included in this study because this subgroup of patients had a relatively more severe clinical status, and the aim was to explore their survival characteristics and to provide a reference for subsequent clinical decision-making. However, this sample selection inevitably introduced a certain degree of selective bias, and the sample sizes of the analyzed groups were small and significantly different, which may have an impact on the statistical efficacy and robustness of the analysis results. Therefore, future studies should consider the inclusion of a broader patient population and more rigorous sampling methods to improve the reliability and generalizability of the findings. Finally, although there was no loss to follow-up, the sample size for the follow-up period was small, and some subgroups had limited patient numbers, which may affect the stability of survival analysis results. Thus, future studies should include more CMM patients and conduct larger-scale longitudinal follow-ups further to validate the mortality rate in CMM patients with POD and assess its clinical significance.

## Conclusion

In summary, this prospective cohort study demonstrated that CMM constitutes an independent risk factor for POD, with significant heterogeneity observed across distinct CMM subgroups. The investigation further revealed that T-tau plays a pivotal mediating role in the underlying pathological mechanism. These findings highlight the critical need for implementing early detection strategies and targeted interventions in high-risk patient populations, which may mitigate POD incidence and improve postoperative survival outcomes.

## Data Availability

The data that support the findings of this study are available in the PNDABLE database. According to relevant regulations, the data could not be shared but could be requested from the corresponding author.

## Research registration unique identifying number (UIN)

The PNDABLE study has received ethical approval from the Ethics Committee of Qingdao Municipal Hospital and is registered with the China Clinical Trial Registry (Registration number: ChiCTR2000033439, registration date: 06/07/2020).
